# New products

**DOI:** 10.1155/S146392469900005X

**Published:** 1999


Journal of Automated Methods & Management in Chemistry, Vol. 21, No. 1 (January–February 1999) pp. 27–32

### New products

### Halogen power transforms moisture analysis for cement additives

Mettler Toledo's new halogen-powered moisture analyser has slashed the drying process for testing samples of concrete additives at Foscroc Ltd, Tamworth, Staffs, from over an hour to a matter of minutes.

The revolutionary design of the model HR73 instrument employs all the power of halogen technology to instantly heat liquid or powdered products to 200°C so that their moisture and solvent content can be measured with precise accuracy and total reproducibility.

The laboratory can now test samples of liquid additives in as little as 3 min, compared with 8 h for a conventional drying oven and at least 70 min using the infrared dryer which it replaces.

Samples are located into a chamber in the body of the instrument resembling that of a CD player and are heated to the required temperature within 30 s—a backlit display showing lab staff the current status and prompting them to the next step before results are recorded on an optional printer unit which is fitted with a calibration kit to provide traceability data for GLP purposes.

Methods for up to 20 substances can be stored in the HR73's databank. The standard interface also allows data to be taken directly from the instrument for analysis on a PC.

The top-of-the-range HR73 model is particularly suitable for product research and development and has an extra 20 g weight range compared with its sister instrument, the HG53 model, which is ideal for more routine measurements in laboratories or production areas.

Unlike other thermogravimetric equipment, both models can be adjusted for weighing performance and operating temperature, enabling instruments in different locations all to be calibrated for consistent results—a first for this method of moisture determination.

*Further information from Roger Norton, Norton Harris Partners, 26 Church Street, Wilmslow, Cheshire SK9 1AU, UK. Tel: 01625 529293; fax: 01625 539737.*

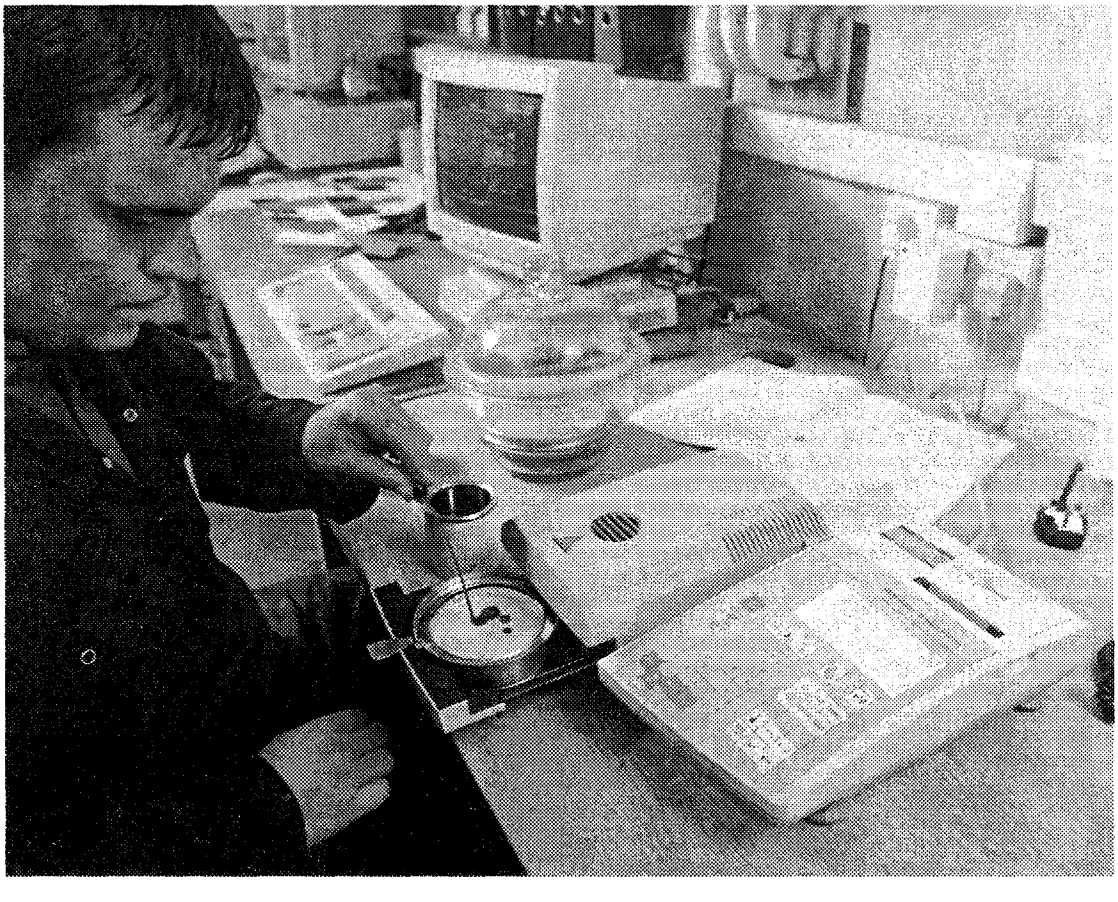

*Fosroc Ltd, Tanworth, Staffs, has dramatically improved its quality control procedures with the aid of this new METTLER TOLEDO halogen-powered moisture analyser which has cut the time previously required to test samples of concrete additives from over an hour to a matter of minutes.*

### ProteinLynx™—Automated protein identification for high-throughput proteomics

Despite its ability rapidly to generate thousands of potential drug candidates, the power of combinatorial chemistry cannot be fully realized until the techniques for massive parallel target identification are on-line. This is the driving force behind proteomics—industrial-scale high-throughput analysis of proteins linked back to biochemical pathways, setting the systematic search for new drug targets at an unprecedented level. Together, these two techniques have the potential to lead to the development of new therapies, greatly reducing health-care costs.

ProteinLynx™ represents the first time that sample management and automated MS data acquisition functions have been combined with client–server bioinformatics to create an integrated single vendor solution. Enabling the mapping of proteins involved in disease mechanisms, proteomics also facilitates the identification of new molecular targets, new diagnostic markers and the development of novel therapeutic agents. This, in practice, requires the isolation and identification of proteins at a rate of thousands per week.

With proteomics emerging as a key new technique in pharmaceutical research, throughput, GALP, security, validation, global system support and commitment to ongoing product development are now regarded as major factors. ProteinLynx™ is implemented on a secure Windows NT ® platform, an extension of Micromass's 'MassLynx' technology.

MALDI-TOF MS workstations for rapid primary mass mapping and electrospray MS-MS workstations for sequence tag determination or *de novo* sequencing are standard analyser options. ProteinLynx™ is compatible with FASTA formatted databases, enabling automated hierarchical search strategies to be implemented on both local and remote servers. Furthermore, the system's open architecture and SampleCentric™ automation facilitate linkage to host bioinformatics systems.

ProteinLynx™ uses a three-tiered analytical strategy for protein identification. The primary screen employs Micromass's TofSpec-2E (MALDI-TOF MS) workstation to conduct automatic peptide-mass fingerprinting of very large arrays of digested protein samples excised from 2D gels. The monoisotopic peptide-mass fingerprints are then automatically matched against dB's of known protein sequences by the ProteinProbe™ search engine. Unmatched proteins from the primary screen may either, at a later date, be flagged for resubmission to the ProteinProbe™ search engine, or, alternatively, automatically scheduled for sequence tagging or *de novo* sequencing by electrospray MS-MS (Q-Tof™).

The secondary screen involves sequence tagging, a powerful approach to identify proteins and peptides unidentified by peptide-mass fingerprinting. Here, a dissolved protein digest sample is analysed using a Q-Tof™ LC-MS-MS workstation, resulting in full product ion spectra being recorded. Subsequent searching may be achieved with ProteinProbe™ as ProteinLynx™ incorporates a 'find sequence tag' tool to generate the 'tag' automatically. Proteins and peptides that remain unidentified by this technique are fully sequenced by ES MS-MS.

The third stage of analysis, *de novo* sequencing, also involves Q-Tof™'s quadrupole-TOF technology, providing high sensitivity performance in the sequencing of sub-femtomole quantities of peptides. PepSeq™, Micromass's new multi-layered peptide sequencing tool, has been designed to take full advantage of high resolution ES MS-MS data from Q-Tof™. Full product ion (MS-MS) spectra are initially processed automatically in three ways:

- charge state de-encryption (MaxEnt-3™)
- identification of C-terminally tagged ions ($^{18}O/^{16}O$)
- sequence tags established

The sequence tag provides the 'cornerstone' from which the complete sequence is built out to cover the C and N termini. Traditional sequencing algorithms 'stall' when they encounter 'gap masses', but PepSeq™ employs LeapLogic™ to jump over 'gaps' and continue sequencing. Gap masses are later rationalized interactively.

MaxEnt-3™ is a proprietary software tool for the 'charge state de-encryption' of MS-MS spectra containing product ions in a range of different charge states. It works by yielding simplified MS-MS spectra, thus facilitating the precise 'reading off' of amino-acid sequences, either manually or automatically with PepSeq™.

*For further information contact Mark McDowall, Micromass UK Limited, Tudor Road, Altrincham, Cheshire WA14 5RZ, UK. Tel.: +44 (0) 161 282 9666; fax +44 (0) 161 282 4400; e-mail: mark.mcdowall@micromass.co.uk*

### Which electrodes for which applications?

Titration has been around for centuries and used in every analytical chemistry laboratory, because of its precision, reproducibility, selectivity and sensitivity... plus the fact that analysis can easily take place on turbid suspensions, emulsions or slurries without sample preparation.

Metrohm have just released a 32-page brochure specifically on electrode systems used for titration, which explains the analysis and specifies which electrodes should be used for a specific application... this is an unique booklet as it applies to all titration products available in the UK.

*For further information please contact Metrohm UK Ltd. Tel. 00 44 (0) 1280 824 824; fax; 00 44 (0) 120824 800; e-mail: enquiry@metrohm.co.uk*

### Automate your chemistry with batches of filter tubes

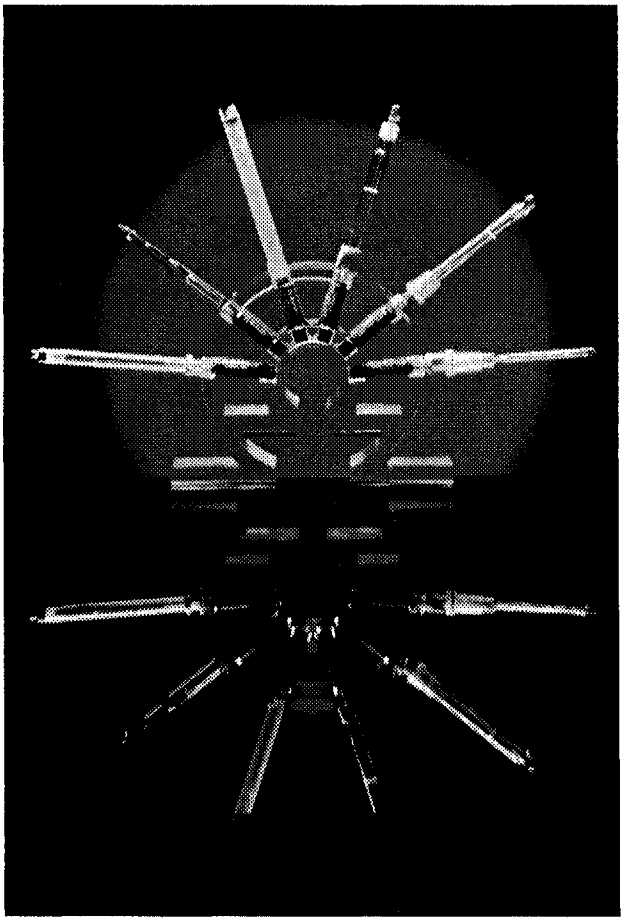

Whatman International has launched an innovative new type of filter tube for aiding batch filtration in combinatorial chemistry techniques. The range is compatible with the Whatman SPE vacuum manifold, as well as other well-proven automated equipment—e.g. the Gilson ASPEC system.

The pigment-free polypropylene filter-tube housing is chemically resistant to a wide spectrum of solvents. Each unit comprises a housing, filter support, and choice of PTFE filter of 0.2, 0.45, 1.0 or 5.0 μm pore size. It is also available with phase separator filter paper. The filter is securely welded to ensure that the filter cannot be bypassed and precious sample lost.

Filter tubes are manufactured to strict quality control standards for guaranteed reliable and reproducible performance under automated conditions. Manual use is also possible, by means of an optional filter tube plunger, and also available are easy-to-use lids and tips making agitation of your sample simple. These are supplied as separate items.

Filter tubes can be supplied in 3, 6 and 12 ml capacities; 100, 50 and 40 units per pack respectively. In addition, alternative sizes and media are available on special order.

*For further information contact Nikki Twining, Whatman International, St Leonard's Road, 20/20 Maidstone, Kent ME16 0LS. Tel.: + 44 (0) 1622 676670; fax: + 44 (0) 1622 677011.*

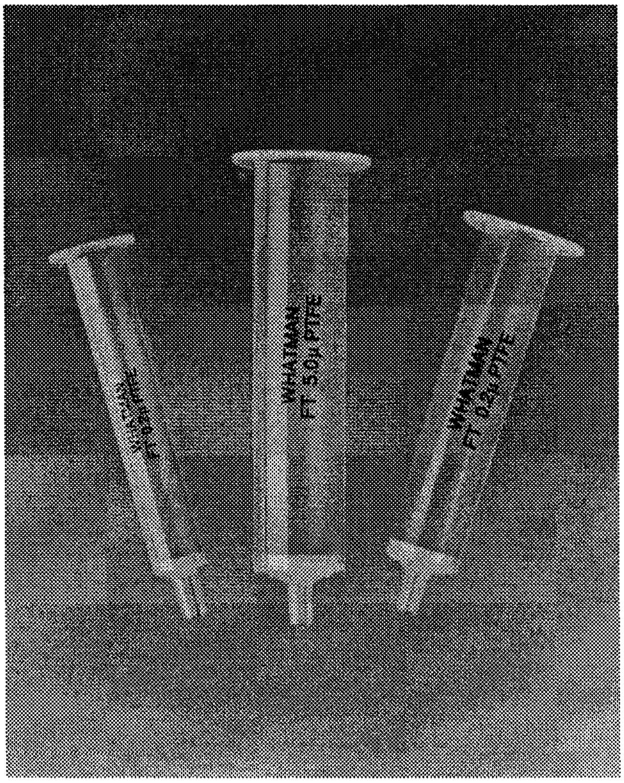

### NanoFlow-Z$^{TM}$: The new, high efficiency electrospray option with the ZSPRAY$^{TM}$ advantage

Having already made a leap in electrospray (ES) inlet technology with the introduction of ZSPRAY$^{TM}$, which features an easily accessible 'open architecture' ion source, Micromass have now developed NanoFlow-Z$^{TM}$ for standard ZSPRAY$^{TM}$ interfaces.

Now the established technique for the analysis of biopolymers, drug metabolites and labile compounds, conventional electrospray systems have been designed for liquid flow rates from a few μl/min up to 1 ml/min, facilitating both flow injection and LC-MS with analytical or microbore HPLC columns.

Electrospray ionization is, however, a concentration dependent technique, with the observed signal intensity almost independent of the flow rate. This fundamental characteristic is exploited by NanoFlow-Z$^{TM}$ for the purpose of obtaining high quality data from electrospraying typically 10–50 nl of solution per minute from a nanovial. As a result, NanoFlow-Z$^{TM}$ yields the same spectral quality as conventional electrospray, but consuming up to 1000 times less sample. In the continuous flow mode the nanovial is replaced with a fused silica capillary where flow rates are typically higher, from 100 to 500 nl/min.

NanoFlow-Z$^{TM}$'s reduced sample consumption means two major benefits:

- *Sensitivity*—e.g. on-column detection limits in the low femtomole range are achievable when packed capillary columns are coupled with a NanoFlow-

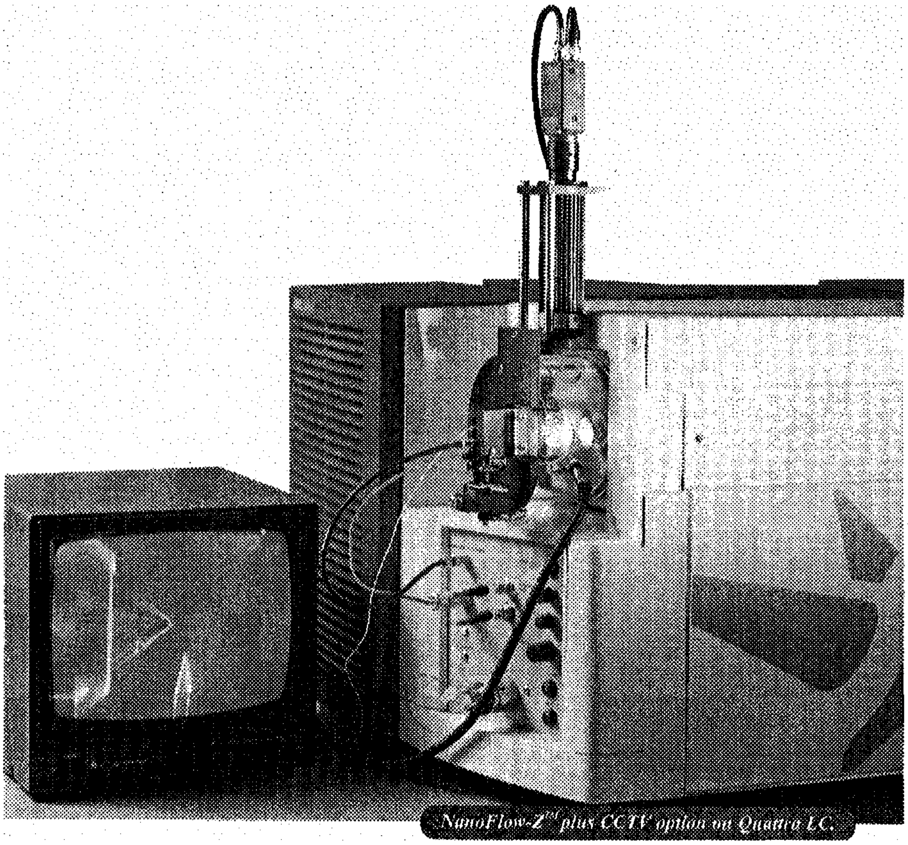

NanoFlow-Z™ plus CCTV option on Quattro LC.

Z™ LC-MS detector. Furthermore, NanoFlow-Z™ is suitable for samples in 100% aqueous to 100% organic mobile phases.
- *Maximum information*—1 µl of a valuable sample solution, loaded into a nanovial, may persist for many tens of minutes, allowing the analyst time to interactively design and automatically execute an extended sequence of experimental procedures. nanoFlow-Z™, when teamed with MS-MS techniques, enables direct characterization of amenable mixtures. Thus, by eliminating the conventional, time-consuming chromatographic separation step, throughput may be significantly increased.

ZSPRAY™ exploits a revolutionary two-stage orthogonal 'Z' sampling technique (patent pending) to deliver the state-of-the-art in contamination avoidance technology, simplicity of operation and enhanced signal to noise.

*Stage one: ruggedness*

The first stage involves the ES aerosol spray being directed perpendicularly past the sampling cone, which is displaced from the central axis of the instrument. Ions are extracted orthogonally from the spray into the sampling cone aperture leaving large droplets, involatile materials, particulates and other unwanted components to collect on a removable baffle plate that screens the vent port. Contaminating deposits will subsequently, after prolonged operation, accumulate on the baffle which can simply be removed and washed clean. The sampling cone will require occasional cleaning to maintain peak performance, but the in-source isolation valve allows easy removal of the cone for cleaning without breaking the vacuum, thus preserving the analytical integrity of the system to maximize throughput.

*Stage two: sensitivity*

This second orthogonal stage enables the volume of gas sampled from atmosphere to be increased by a factor of 4 in ZSPRAY™ compared with conventional API sources. A freely expanding jet, representing a region of high pressure compared to the surrounding vacuum, is formed when gas at atmospheric pressure is sampled through an aperture into a partial vacuum. This jet, when directed into the second aperture of a conventional API interface, increases the flow of gas through the second aperture. Thus, maintaining a suitable vacuum in the MS analyser places a restriction upon the maximum diameter of the apertures in such an on-axis interface. If, however, the jet passes orthogonal to the second aperture, the flow into it is significantly decreased. Consequently, in the ZSPRAY™ interface the sampling cone aperture's diameter may be increased to allow four times as much gas to be sampled from the atmospheric spray without degrading the analyser vacuum or increasing the pumping speed of the system. This increased gas conductance delivers an observable gain in ion current of a factor of 2. In the partial vacuum of the ion block, ions are extracted electrostatically into the hexapole ion bridge which efficiently transports ions to the analyser. Additionally, orthogonal sampling significantly reduces background or 'neutral' noise by contributing to the exceptional limits of detection observed with ZSPRAY™ coupled with Micromass's quadrupole or orthogonal acceleration time-of-flight (oa-TOF) mass analysers.

A standard ZSPRAY™ ion source is simply converted to NanoFlow-Z™ operation replacing the conventional electrospray probe and alignment mechanism with a NanoFlow-Z™ probe and high precision (X, Y, Z) manipulator stage. The NanoFlow-Z™ inlet features a microscope and fibre-optic illumination, allowing detailed viewing of capillary tips *in situ*.

The continuous flow option has been designed for both flow injection analysis and on-line LC-MS with packed fused silica columns, and has been optimized for flow rates in the 100 nl/min to > 500 nl/min range. Samples for off-line analysis are typically injected through a valve loop (1 μL) injector, and are pumped along the probe through a fused silica tube connected to the nebulizer by an ultra-low DV, stainless steel union.

Providing the ultimate in sensitivity, the Nanoflow-Z™ probe also enables real-time micro-reaction monitoring and direct analysis of unseparated peptide mixtures. Optionally available in either continuous flow or nano-vial configurations for the new Platform-LCZ™ (marketed by Waters Corporation), Quattro-LC™, LCT™ and Q-Tof™ instruments ordered with standard ZSPRAY™ ion sources, NanoFlow-Z™ is also available in upgrade packages for previously installed instruments already equipped with ZSPRAY™ electrospray ion sources.

*For further information contact Steve Preece, Micromass UK Limited, Tudor Road, Altrincham, Cheshire WA14 5RZ, UK. Tel.:@ +44 (0) 161 282 9666; fax: +44 (0) 161 282 4400; e-mail: steve.preece@micromass.co.uk*

### 'The TitrIC system'

The TitrIC system from Metrohm combines the techniques of Metrohm titration with their ion chromatography system, for water testing and environmental labs. These two techniques are linked together through the 730 Autosampler.

The TitrIC autosampler can accommodate 136 samples. First the conductivity of the sample is measured, then a portion of the sample is taken to the 733/732 ion chromatography system for anionic analysis, i.e. F, Cl, Br, I, $NO_2$, $NO_3$, $SO_4$, etc. Meanwhile the 730/736/Ti net titration system analyses the pH, P + M, alkalinity and Ca/Mg hardness. Within 15 min all the parameters are linked together in a final report. The systems are cleaned automatically so they are immediately ready for the next sample.

*For further information, please contact Metrohm UK Ltd, Unit 2, Top Angel, Buckingham Industrial Park, Buckingham MK18 1TH, UK. Tel.: + 44 (0) 1280 824 824; fax: + 44 (0) 1280 824 800; e-mail: TechSupp@www.metrohm.co.uk/*

### Automated dissolution testing

A new dissolution capabilities brochure is now available. This brochure highlights the MultiDose and MultiDose Plus Workstations as productivity enhancing tools for fully automating dissolution testing. It discusses the most common reasons for implementing automation in pharmaceutical dosage form testing areas: to reduce the cost of testing, to reduce cycle time, to improve the quality of results, to simplify technology transfers and to control outsourcing costs. An explanation of how the workstations help solve these specific problems is provided.

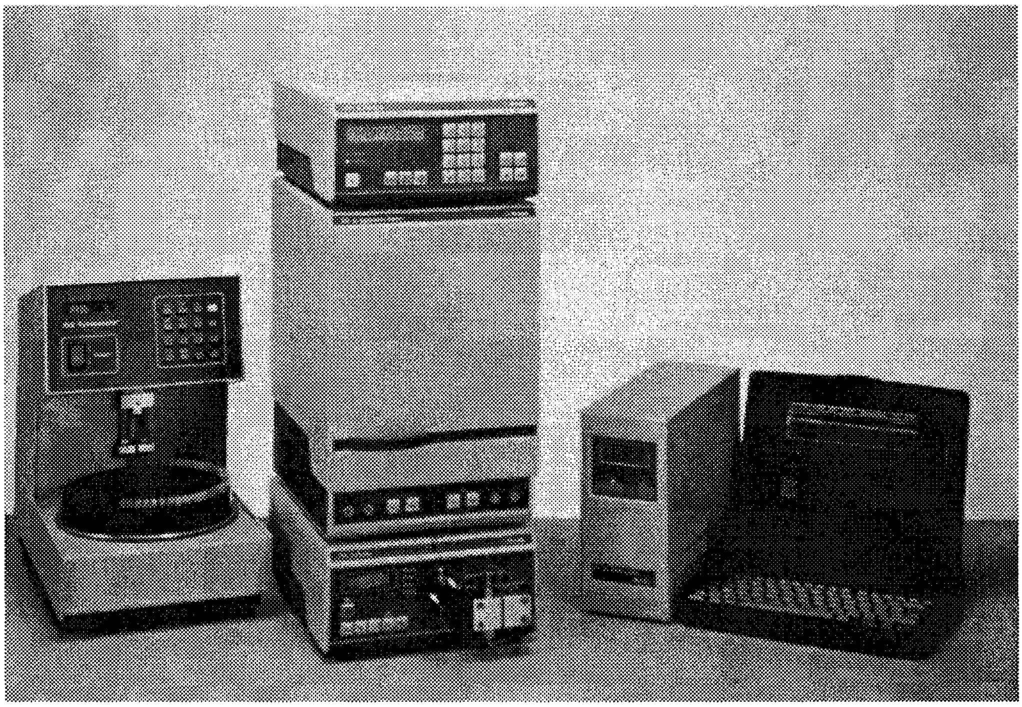

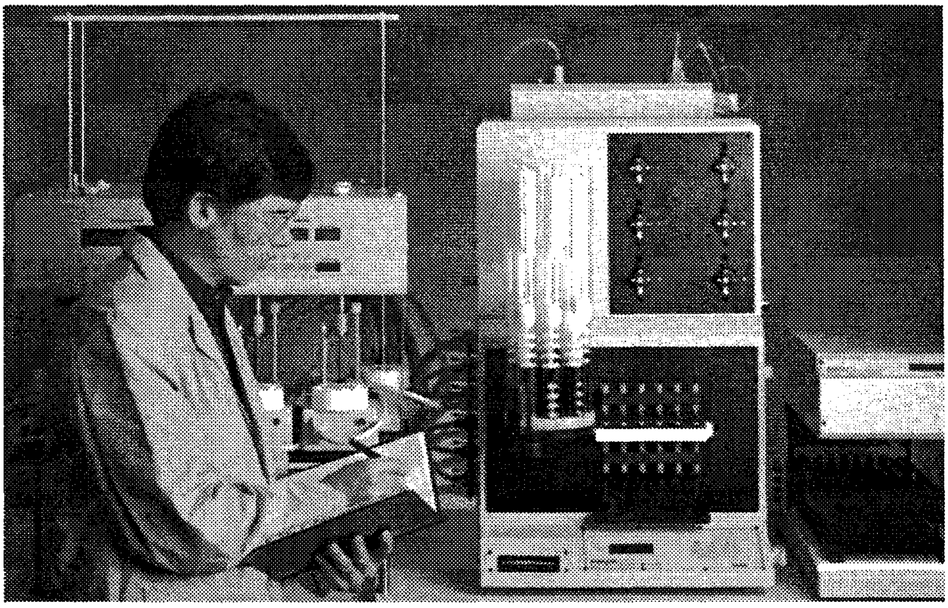

*Productivity enhancing workstation for fully automated dissolution testing.*

A treatment of the salient features and benefits of the automation technology is shown in tabular form.

The MultiDose Plus, in conjunction with an 'automation ready' VanKel 7000 dissolution apparatus, can run up to eight batches unattended. The system preheats and conditions the dissolution media, fills each vessel with the appropriate volume of media, verifies temperature, starts the dissolution test, removes sample aliquots at the user-defined time interval(s), then empties and cleans each vessel before starting another run. Dissolution samples are either stored for off-line analysis or can be analysed on-line with an appropriate multi-cell UV/VIS spectrophotometer. All operations of the system are documented in an electronic audit trail.

*For more information contact: Sharon Correia, Zymark Corporation, Zymark Center, Hopkinton, MA 01748, USA; Tel.: +1 508 497 6403; fax: +1 508 435 3439; e-mail: sharon.correia@zymark.com*

